# A novel diet‐induced murine model of steatohepatitis with fibrosis for screening and evaluation of drug candidates for nonalcoholic steatohepatitis

**DOI:** 10.14814/phy2.13016

**Published:** 2016-11-08

**Authors:** Chieko Ejima, Haruna Kuroda, Sonoko Ishizaki

**Affiliations:** ^1^ Research InstituteEA Pharma Co. Ltd.KanagawaJapan

**Keywords:** Animal model, branched‐chain amino acids, Nonalcoholic steatohepatitis, screening

## Abstract

Many animal models of nonalcoholic steatohepatitis have been reported. While these models exhibit mild onset of hepatitis and fibrosis, induction is often slow. For faster screening of drug candidates, there is a compelling need for convenient animal models of steatohepatitis and nonalcoholic steatohepatitis in which fatty liver and hepatitis are stably induced within a short period. Here, we analyzed the hepatic lipid composition in nonalcoholic steatohepatitis, and used this information to successfully establish a murine model where steatohepatitis is induced within only 1 week using a novel diet (steatohepatitis‐inducing high‐fat diet, STHD‐01) high in saturated fatty acids and cholesterol. After receiving STHD‐01 for 1 week, normal mice (C57BL/6J) showed elevated markers of fatty liver and hepatitis, including hepatic triglycerides and plasma alanine aminotransferase; the administration of angiotensin receptor blockers reduced these symptoms. Furthermore, we confirmed that STHD‐01 administration for 36 weeks induced not only sustained elevation of hepatic triglyceride and plasma alanine aminotransferase levels, but also fibrosis and tumor formation. Pretreatment with the carcinogen diethylnitrosamine accelerated tumor formation, and hepatic lesions were observed within 30 weeks of STHD‐01 feeding following diethylnitrosamine pretreatment. Finally, branched‐chain amino acids, known to reduce the risk for hepatocellular carcinoma in preclinical models, were effective in reducing the progression of liver fibrosis induced by STHD‐01 feeding after diethylnitrosamine pretreatment. We concluded that STHD‐01 administration successfully induces steatohepatitis within a short period of time. The proposed murine model is suitable for studying the long‐term effects of pharmaceutical agents targeting steatohepatitis, fibrosis, and tumor formation.

## Introduction

Nonalcoholic steatohepatitis (NASH) is pathologically characterized by progression from simple steatosis to steatohepatitis without a definite history of alcohol intake or hepatitis virus infection, and can culminate in hepatic carcinogenesis due to fibrosis and cirrhosis (Michelotti et al. 2013; Ahmed et al. 2015; Spengler and Loomba 2015). Although many animal models have been proposed, the unavailability of robust NASH animal models with short evaluation periods delays the development of drugs for this disease. Examples of currently available fatty liver disease models include mice fed with a methionine‐ and choline‐deficient diet (MCD), genetically modified animals (such as ob/ob mice, db/db mice, and melanocortin‐4 receptor‐deficient mice), and forced overfeeding models (Sahai et al. 2004; Deng et al. 2005; Varela‐Rey et al. 2009; Hebbard and George 2011; Itoh et al. 2011; Takahashi et al. 2012; Imajo et al. 2013; Sanches et al. 2015). Among these existing models, MCD‐fed mice have been widely used as NASH models, although they exhibit a significant decline in body weight. To overcome this limitation, a new experimental diet for inducing NASH was recently introduced; it is based on adjusted methionine and high fat content, and minimizes the loss of body‐weight (Matsumoto et al. 2013).

Meanwhile, there are increasing reports of NASH animal models where symptoms are induced by excessive ad libitum fat loading, including a model of steatohepatitis exacerbated by providing a high‐fat diet with or without cholesterol in normal or genetically modified animals (Matsuzawa et al. 2007; Yetti et al. 2013; Ichimura et al. 2015; Machado et al. 2015). The STAM^®^ NASH mouse model produces inflammation, fibrosis, and (frequently) hepatic carcinogenesis in rodents (Takakura et al. 2014). While these models may indeed reflect the actual clinical condition, they are not suitable for in vivo screening because of slow disease induction.

A previous report showed that cells are protected from lipotoxicity after excessive lipid intake by conversion of lipotoxicity‐inducing lipids to triglycerides (TG) and storage within the adipose tissue (Listenberger et al. 2003). To induce steatohepatitis in a shorter time, we focused on saturated fatty acids that are well known for their potent lipotoxicity. In patients with NASH, the saturated fatty acid and free cholesterol content of the liver are significantly higher than in healthy individuals and NAFLD patients (Puri et al. 2007). Furthermore, free cholesterol accumulation in hepatic stellate cells is reported to sensitize these cells to transforming growth factor‐beta (TGF‐*β*)‐induced activation in a vicious cycle, leading to exaggerated liver fibrosis in NASH (Tomita et al. 2014). Dietary cholesterol appears to confer a “second hit” that results in a distinct hepatic phenotype characterized by increased inflammation and oxidative stress (Subramanian et al. 2011). Therefore, we prepared an experimental diet containing a plentiful amount of saturated fatty acids and cholesterol to induce NASH in mice. We referred to this experimental diet as the steatohepatitis‐inducing high‐fat diet (STHD‐01), and investigated whether it can induce fatty liver and hepatitis within a short period of time.

In this study, we examined whether clinically effective drugs such as telmisartan, olmesartan, vitamin E, and ezetimibe are useful against the changes induced by short‐term feeding of STHD‐01 in mice. Moreover, we aimed to hasten the development of fibrosis due to fatty liver and steatohepatitis in order to evaluate the effect of branched‐chain amino acids (BCAAs), which are reported to alleviate hypoalbuminemia and improve the incidence of severe events in patients with decompensated hepatic cirrhosis (Muto et al. 2005). To that end, we devised an STHD‐01 long‐term feeding model (30 weeks) after pretreatment with a hepatocarcinogenic agent, diethylnitrosamine (DEN), which is widely used for inducing liver tumors (Tolba et al. 2015).

## Materials and Methods

### Animals

Male C57BL/6J mice (Sankyo Labo Service, Tokyo, Japan) were housed in a temperature‐controlled breeding room at 20–26°C with a 12:12‐h light (22:00 to 10:00)/dark (10:00 to 22:00) cycle. The animals had access to CRF‐1 solid feed (Oriental East Co., Tokyo, Japan) and drinking water ad libitum during the quarantine period. During the study period, control mice were fed a standard diet (SD) based on the AIN‐93G formulation, whereas experimental mice were fed an STHD‐01 formulation containing 40% fat and 5% cholesterol, ad libitum. Each diet was supplemented with equal quantities of proteins, sucrose, minerals, vitamins, and choline. The fatty acid composition of the STHD‐01 is provided in Table 1. All studies were approved by the ethics committee of the Research Institute of Ajinomoto Pharmaceuticals Co. Ltd., the forerunner of EA Pharma Co. Ltd., in accordance with their rules of ethics for animal research.

**Table 1:** Fatty acid composition of the standard diet (SD) and of a steatohepatitis‐inducing high‐fat diet (STHD‐01) developed in the present study

Fatty acid	SD	STHD‐01
14:0	0.2%	–
16:0	12.3%	24.0%
16:1	–	0.2%
17:0	–	0.2%
18:0	4.2%	33.0%
18:1	24.3%	32.1%
18:2n‐6	50.7%	8.2%
18:3n‐3	6.4%	1.0%
20:0	0.4%	1.1%
20:1	0.2%	–
22:0	0.3%	0.2%
24:0	0.2%	–
Unknown	0.8%	–

Each fatty acid is identified by the number of carbon atoms in the chain and the total number of unsaturated bonds, together with the position of the unsaturated bonds counting from the terminal methyl carbon (where necessary). All values were measured by gas chromatography and are presented as percentage of mass.

### STHD‐01 feeding and treatment in mice

Short‐term feeding: For the observation of short‐term pathophysiological profiles in control and experimental mice, plasma alanine aminotransferase (ALT) and hepatic TG levels were determined on days 1, 3, 6, and 10. We used the STHD‐01 short‐term (6‐day) feeding model to evaluate the efficacy of several drugs previously reported to counteract the symptoms of NASH. The following drugs were mixed with the STHD‐01 feed to test their ability to alleviate hepatitis: telmisartan, 5 mg/kg equivalent (Micardis^®^, Astellas Pharma, Tokyo, Japan); olmesartan medoxomil, 3 mg/kg equivalent (Olmetec^®^, Daiichi Sankyo Pharma, Tokyo, Japan); vitamin E, 500 mg/kg equivalent; 300 mg/capsule d‐*α*‐tocopherol (Kokando Pharma, Hyogo, Japan); ezetimibe, 10 mg/kg equivalent (Zetia^®^, Schering‐Plough, Kenilworth, NJ); obeticholic acid, 10 mg/kg equivalent (6‐ECDCA/INT‐747, Enzo Life Sciences, New York, NY); eicosapentaenoic acid (EPA), 4000 mg/kg equivalent (Epadel^®^, Mochida pharmaceutical, Tokyo, Japan); pentoxifylline, 50 mg/kg equivalent (Sigma‐Aldrich, St. Louis, MO); pioglitazone hydrochloride, 10 mg/kg equivalent (ChemPacific, Baltimore, MD); ursodeoxycholic acid, 360 mg/kg equivalent (Sigma‐Aldrich); BCAAs, 4200 mg/kg equivalent (Livact^®^ composition, Ajinomoto, Tokyo, Japan). As the drugs were mixed with the chow, the amount of drug ingested (i.e., real dose) depended on the food intake of each animal. For the purpose of our analysis, the amount of each drug consumed by the animals was calculated based on the average food intake. Rolipram at a dosage of 30 mg/kg (A.G. Scientific, Inc., San Diego, CA) was administered subcutaneously.

Long‐term feeding: To determine whether long‐term STHD‐01 feeding can induce liver fibrosis and tumor formation, and whether pretreatment with the carcinogen DEN accelerates the process, C57BL/6J mice were fed with STHD‐01 for 36 weeks, or for 30 weeks with normal drinking water after receiving water containing 0.04% DEN for 10 days prior to initiating STHD‐01 feeding (Ishizaki et al. 2013). Plasma ALT and hepatic TG were measured at the end of the STHD‐01 feeding period. In mice receiving long‐term STHD‐01 feeding after DEN pretreatment, STHD‐01 was mixed with 3% BCAAs (L‐isoleucine:L‐leucine:L‐valine at ratios of 1:2:1.2, Ajinomoto, Tokyo, Japan) to examine their effect on liver fibrosis. In the control group, STHD‐01 was mixed with 3% casein instead of BCAAs.

### Determination of plasma ALT, hepatic TG, and hepatic total cholesterol levels

Plasma ALT was determined using a Fuji Dri‐Chem FDC7000V device (Fuji Film, Tokyo, Japan). To determine hepatic TG and total cholesterol (TCHO) levels, samples were obtained using the methods of Folch et al. 1957. TG and TCHO levels were determined using a TG E test kit and cholesterol E test kit, respectively (Wako Pure Chemical Industries, Osaka, Japan).

### Histology and digital image analysis

Liver tissues were stained with hematoxylin and eosin. Liver fibers were stained with Sirius red. For quantitation of liver fibrosis, 5 images each of Sirius red‐stained liver samples were obtained under a bright‐field microscope. After binarizing the bright‐field microscopic images of Sirius red‐stained samples into positive and negative using dedicated software (WinROOF, Ver. 5.7.2, Mitani Corp., Tokyo, Japan), we calculated the proportion of the positive area.

### RNA extraction and gene expression analysis

We performed RT‐PCR with liver samples from mice fed for 36 weeks or for 30 weeks after DEN pretreatment. Commercial kits were used to extract total RNA in the liver and to synthesize cDNA (TRIzol^®^ Plus RNA Purification Kit and SuperScript^®^ VILO™ cDNA Synthesis Kit, Life Technologies, Carlsbad, CA). Levels of gene expression were measured with Power SYBR^®^ Green Master Mix (Life Technologies) and a 7500 Fast Real‐Time PCR System (Applied Biosystems, Foster City, CA). All gene expression levels were normalized to that of *Gapdh*. The primer sequences are listed in the Supplemental Table, available online at the Journal's website.

### Isolation of nonparenchymal cells from the liver

To characterize the effects of STHD‐01 feeding on hepatic nonparenchymal cells (NPCs, including liver sinusoidal endothelial cells and Kupffer cells), we investigated tumor necrosis factor (TNF) production in response to lipopolysaccharide (LPS, *Escherichia coli* 055:B5; Sigma‐Aldrich) treatment of NPCs isolated from the livers of mice fed for 1 or 8 weeks with STHD‐01. Hepatic NPCs were prepared with the gentleMACS Dissociator (Miltenyi Biotec GmbH, Bergisch Gladbach, Germany) using the manufacturer's recommended program settings. Specifically, liver tissues were ground coarsely with prewarmed dissociation mix (20 μL of 0.5 mol/L CaCl_2_ solution), 500 collagen digestion units per mL of collagenase IV (Sigma‐Aldrich) solution, and 150 U/mL of DNase I (AppliChem, Darmstadt, Germany) solution in 4.4 mL Krebs‐Ringer‐Buffer in a gentleMACS C Tube (Miltenyi Biotec GmbH). To eliminate red blood cells, density gradient centrifugation by Histodenz (Sigma‐Aldrich) was used. The cell density was adjusted to 1.0 × 10^6^ cells/mL, and isolated NPCs were seeded into 96‐well plates (100 μL/well, Thermo Scientific Nunc; Thermo Fisher Scientific Inc., Waltham, MA) and maintained in RPMI 1640 (Thermo Scientific Gibco; Thermo Fisher Scientific Inc.) supplemented with 10% FBS (low‐endotoxin; Tissue Culture Biologicals, Tulare, CA) and 1% streptomycin/penicillin (Life Technologies) at 37°C in a 5% CO_2_/95% air environment overnight. Next, each well was washed and LPS was added for 22–24 h prior to measurement of TNF‐*α* by ELISA (Quantikine ELISA, Mouse TNF‐*α*; R&D Systems, Minneapolis, MN); the final LPS concentrations were 0, 10, 100, and 1000 ng/mL in Eagle's basal medium (Thermo Scientific Gibco; Thermo Fisher Scientific Inc.) supplemented with 0.5% bovine serum albumin (Fraction V, fatty acid free and low endotoxin; MP Biomedicals, Irvine, CA).

### Statistical analysis

Results are expressed as means ± standard errors. Statistical analyses were performed with the EXSUS or JMP 10 (The SAS System for Windows Ver. 9.3, SAS Institute Inc., Cary, NC). Student's *t*‐test was used to compare the STHD‐01 and SD groups. The statistical significance of the effect of drug candidates was evaluated by Dunnett's test. *P *<* *0.05 was considered significant.

## Results

### Short‐term feeding with STHD‐01

**Figure 1:**
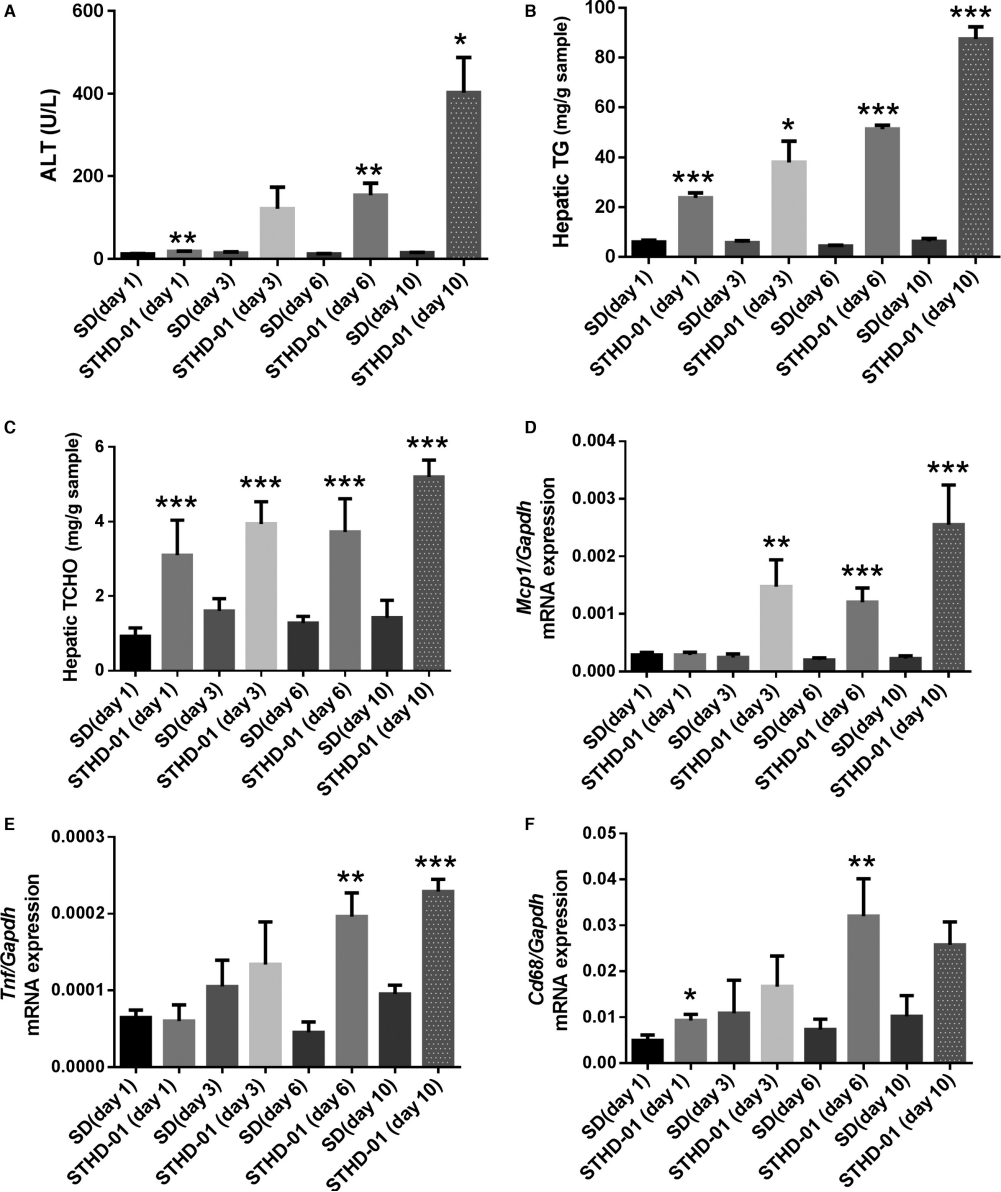
Alanine aminotransferase (ALT; hepatitis), hepatic triglycerides (TG; fat accumulation in the liver), hepatic cholesterol (TCHO), and gene expression levels in the livers of mice fed with steatohepatitis‐inducing high‐fat diet (STHD‐01). ALT (A), hepatic TG (B), and hepatic TCHO (C) levels, as well as the expression levels of *Mcp1* (D), *Tnf* (E), and *Cd68* (F) in the liver were determined after feeding with standard diet (SD) or STHD‐01 for 1, 3, 6, and 10 days. At each time point, *n* = 4–5 in each group (SD and STHD‐01. Bars represent the means ± standard errors. *P < 0.05, **P < 0.01, ***P < 0.001 compared with the SD group (at each time point, Student's *t*‐test).

On day 1, the mean ALT level in the STHD‐01 group was significantly higher than that in the SD group (18.2 ± 1.2 U/L vs. 12.2 ± 1.0 U/L, respectively, *P *<* *0.01), as shown in Figure 1A. Additionally, the mean ALT levels in the STHD‐01 group were significantly higher than those in the SD group on both day 6 and day 10. Hepatic TG and TCHO had substantially accumulated on day 1 and increased at subsequent time points during STHD‐01 feeding (Fig. 1B and C). Hepatic gene expression analysis showed a significant increase in *Mcp1* on day 3, followed by increased gene expression of *Tnf* and *Cd68* (Fig. 1D–F).

### Long‐term feeding with STHD‐01

**Figure 2:**
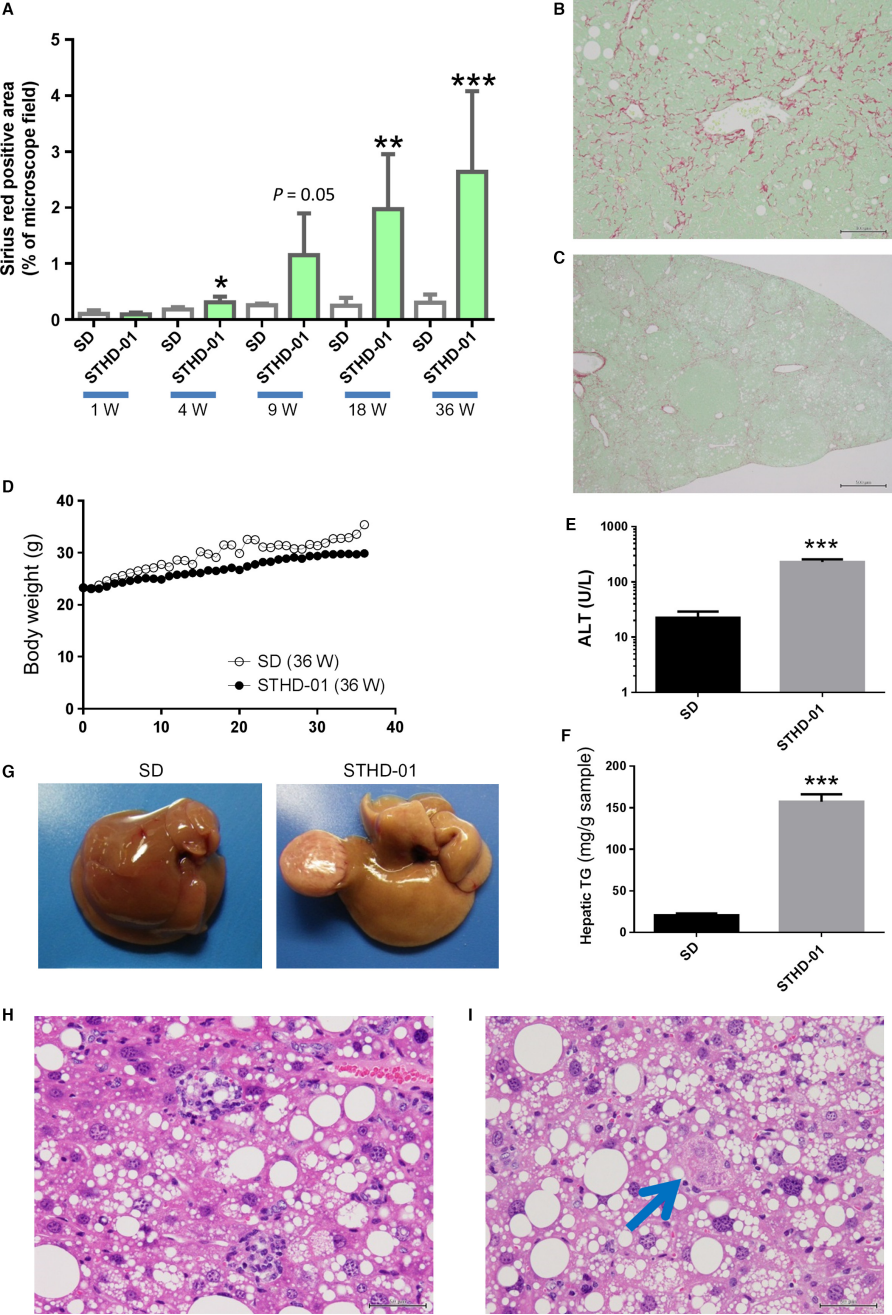
Changes in mice fed with steatohepatitis‐inducing high‐fat diet (STHD‐01). (A) Percentage of areas positive for Sirius red staining in the livers of mice fed with STHD‐01 for 1 week (*n* = 3–4), 4 weeks (*n* = 3–4), 9 weeks (*n* = 4–5), 18 weeks (*n* = 5) and 36 weeks (*n* = 7–9). Sirius red staining of a liver from a mouse‐fed STHD‐01 for 36 weeks (B and C). Perivenular‐pericellular fibrosis and a slender, chicken‐wire pattern (B, ×200); and extensive fibrosis over the entire liver (C, ×40) were noted. Changes in body weight (D), alanine aminotransferase levels (E), hepatic triglyceride levels (F), and macroscopic findings of the liver (G) in mice fed with STHD‐01 for 36 weeks. Hematoxylin and eosin‐stained images of liver tissues in mice‐fed STHD‐01 for 36 weeks (H and I). Large lipid droplets and lobular inflammation (H) and Mallory–Denk body (I) were noted. Bars represent the means ± standard errors. *P < 0.05, **P < 0.01, ***P < 0.001 compared to the SD group (Student's *t*‐test).

Liver areas positive for Sirius red staining in mice fed with STHD‐01 throughout the study period gradually increased (Fig. 2A). Furthermore, Sirius red staining of the liver from STHD‐01‐fed mice showed positive findings indicative of perivenular‐pericellular fibrosis and slender, chicken‐wire patterns characteristic of clinical NASH (Fig. 2B). Extensive fibrosis occurred in the entire liver, although no rigid bridging fibrosis was observed (Fig. 2C). Body‐weight gain tended to be suppressed more in the STHD‐01 group than in the SD group (Fig. 2D). No animal presented with extreme body‐weight loss, which is a feature of the MCD model. We observed continuous elevation in ALT and TG accumulation in the liver of mice‐fed STHD‐01 (Fig. 2E and F). Hepatic TG levels were highest in mice‐fed STHD‐01 for 36 weeks without DEN pretreatment. Necropsy showed no remarkable macroscopic changes in the livers of the SD‐fed animals; however, in mice‐fed STHD‐01 for 36 weeks, macroscopic findings indicated definite fatty liver in all animals, and tumor formation in six of the nine animals (Fig. 2G). The tumors in these six animals were benign adenomas. In mice‐fed STHD‐01 for 36 weeks, histological findings included characteristic features of NASH, such as macro‐microvesicular steatosis and lobular inflammation (Fig. 2H), as well as Mallory–Denk bodies (Fig. 2I).

**Figure 3:**
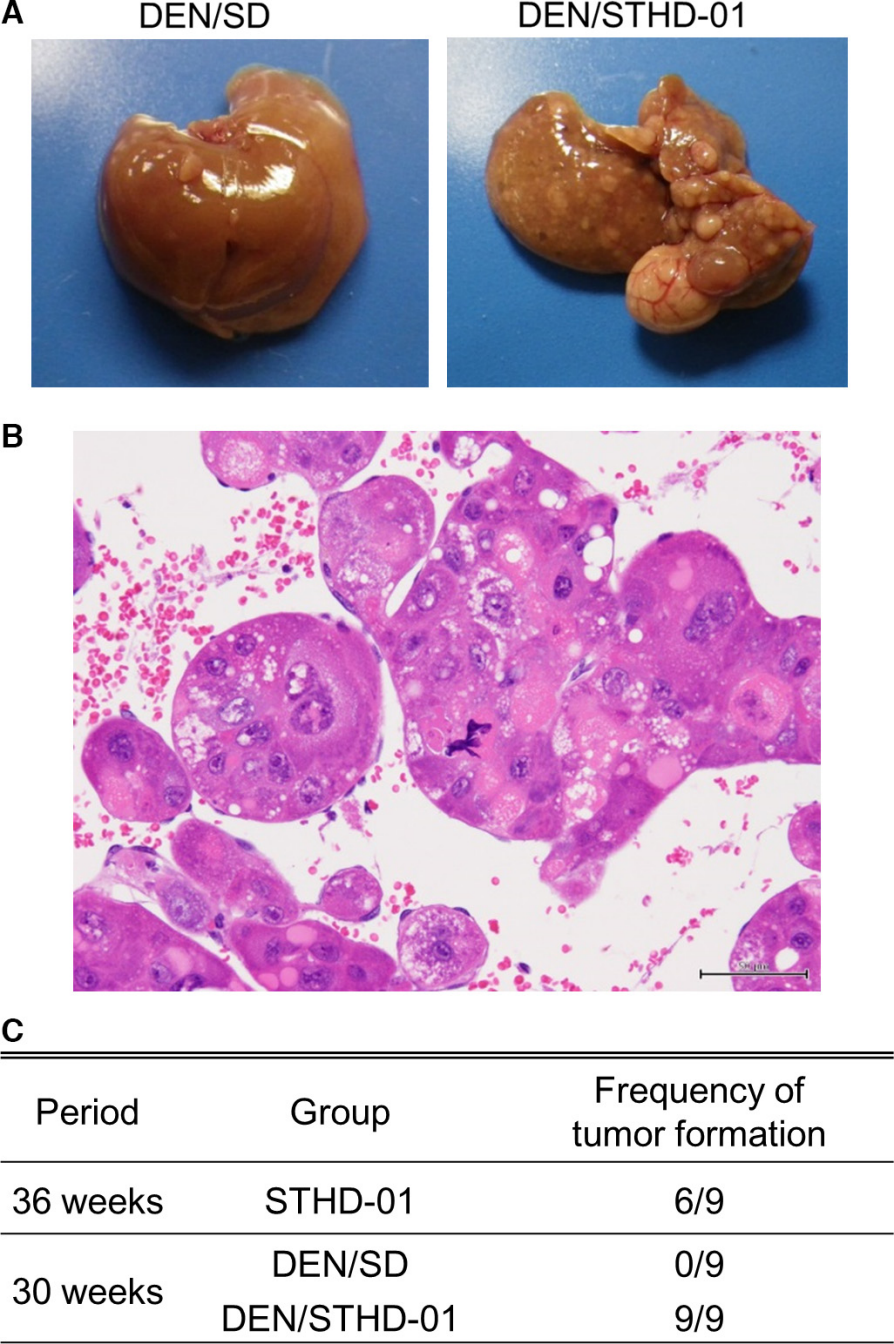
Macroscopic and histopathological findings in mice fed with steatohepatitis‐inducing high‐fat diet (STHD‐01) for 30 weeks after diethylnitrosamine (DEN) pretreatment. Macroscopic findings in the liver of mice fed with standard diet (SD) or STHD‐01 for 30 weeks immediately after switching from drinking water containing 0.04% DEN for 10 days to normal drinking water (A). Histopathological findings in the liver. Hepatocellular carcinoma with structural and cellular atypia in a mouse fed with STHD‐01 for 30 weeks after DEN pretreatment (B). Frequency of tumor formation (C).

Tumor formation was evident in all mice‐fed STHD‐01 for 30 weeks after DEN pretreatment (Fig. 3A). In DEN‐pretreated mice, the liver was slightly reddish compared to that in mice without DEN pretreatment, and exhibited the characteristic formation of large tumors and scattered small tumors. One DEN‐pretreated STHD‐01‐fed mouse had tumors with structural and cellular atypia, confirming the presence of hepatocellular carcinoma (Fig. 3B). Meanwhile, tumor formation was not observed in mice fed with SD for 30 weeks after DEN‐pretreatment (Fig. 3C). These results showed that STHD‐01 augmented the tumorigenic potential of DEN.

### Effects of STHD‐01 on mice

**Figure 4:**
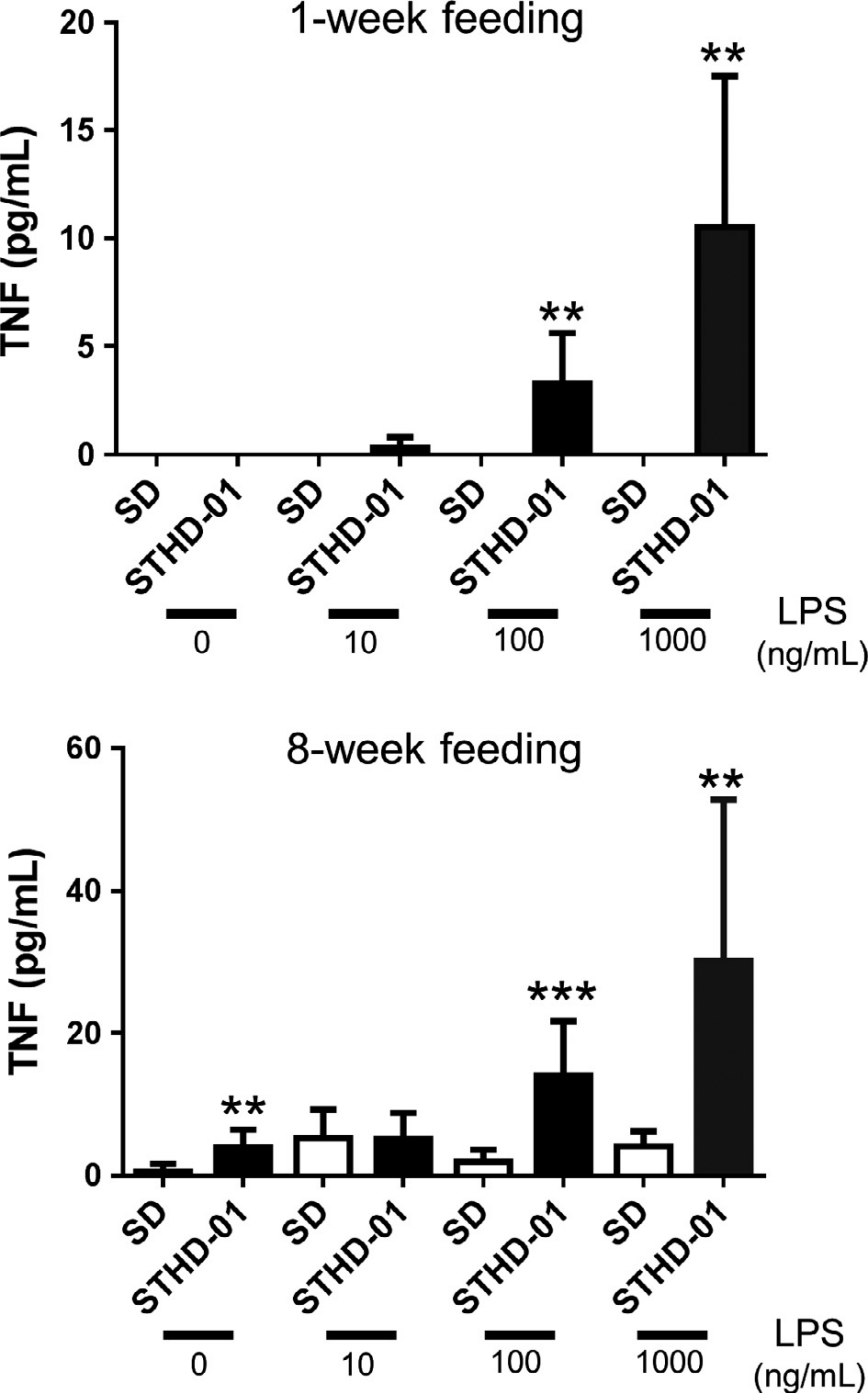
Tumor necrosis factor (TNF) production in response to lipopolysaccharide (LPS) stress of isolated hepatic nonparenchymal cells (NPCs) from livers of mice fed with steatohepatitis‐inducing high‐fat diet (STHD‐01) for 1 week (A, *n* = 3) or 8 weeks (B, *n* = 4). Bars represent the means ± standard errors. **P < 0.01, ***P < 0.001 compared to the SD group (at each concentration, Student's *t*‐test).

STHD‐01 increased the expression of various genes involved in inflammation (*Tnf*,* Mcp1*,* Il6*,* Spp1*/osteopontin), fibrosis (*Col1a1*,* Acta2*/*α*‐SMA, *Timp1*), premalignant lesion formation (*Epcam*,* Krt8*/Cytokeratin8), and stress (*Nrf2*,* Eif2ak3*/Perk, *Ddit3*/chop) in both mouse models (Table 2). Expression of *Cyp7a1*,* Nrf2*,* Trp73*/p73, *Sema7A,* and *Il17* genes increased over twofold in mice‐fed STHD‐01 for 30 weeks after DEN pretreatment compared to that in mice fed for 36 weeks without pretreatment (*Cyp7a1*: 2.6‐fold, *Nrf2*: 2.8‐fold, *Trp73*/p73: 2.6‐fold, *Sema7A*: 2.0‐fold, *Il17*: 2.1‐fold,). We postulate that DEN pretreatment coupled with a long‐term STHD‐01 triggers changes in cholesterol metabolism and in the population of liver immune cells. STHD‐01 induced higher TNF production by hepatic NPCs than that induced by SD upon NPC stimulation with 100 or 1000 ng/mL of LPS (Fig. 4).

**Table 2:** Changes in gene expression levels in the livers of mice fed with a steatohepatitis‐inducing high‐fat diet (STHD‐01) for 36 weeks, or for 30 weeks after diethylnitrosamine (DEN) pretreatment

Gene name	36‐week feeding		30‐week feeding after DEN pretreatment	
	STHD‐01/SD	*P‐*value	STHD‐01/SD	*P‐*value
Inflammation				
* Tnf*	5.8	0.0008	8.0	0.0001
* Mcp1*	8.2	0.0009	7.2	0.0005
* Il6*	2.2	0.0001	3.3	0.0009
* Spp1* (osteopontin)	9.3	0.0006	12.1	0.0003
* Adgre1* (F4/80)	2.8	0.0001	2.6	0.0006
* Il17*	1.4	0.0386	2.9	0.0020
* Nos2*	6.8	0.0020	12.9	0.0002
Fibrosis				
* Col1a1*	27.4	0.0001	15.2	0.0001
* Acta2 (alpha SMA)*	4.7	0.0004	3.3	0.0002
* Serpinh1* (HSP47)	4.2	0.0002	4.8	0.0001
* Timp1*	27.4	0.0001	24.7	0.0001
* Tgfb1*	2.3	0.0001	2.5	0.0002
Premalignant lesions				
* Epcam*	14.1	0.0013	18.5	0.0008
* Krt8* (Cytokeratin8)	4.4	0.0001	3.9	0.0003
* Ctnnb1* (beta‐Catenin)	1.5	0.0001	1.8	0.0011
* Axin2* (conductin)	2.3	0.0367	2.9	0.0032
Oxidative stress				
* Nrf2*	1.7	0.0000	4.8	0.0001
* Hmox1*	1.8	0.0002	2.5	0.0187
Endoplasmic reticulum stress				
* Atf4*	1.8	0.0393	1.7	0.0033
* Eif2ak3*(Perk)	2.7	0.0001	4.8	0.0005
* Hspa5* (Grp78)	1.3	0.0598	1.6	0.0333
* Ddit3* (chop)	1.9	0.0042	3.4	0.0001
* Xbp1*	1.3	0.0435	1.6	0.0033
* Traf2*	2.2	0.0001	2.4	0.0001
Metabolism				
* Cyp7a1*	1.5	0.1650	3.9	0.0001
* Phgdh*	1.9	0.0251	3.3	0.0026
* Pparg*	2.6	0.0000	4.2	0.0001
* Mthfd2*	4.6	0.0033	4.5	0.0003
* Ucp2*	3.7	0.0001	4.0	0.0001
* Fgf21*	5.8	0.0948	4.3	0.0286
Other genes				
* Trp73* (p73)	1.7	0.1805	4.5	0.0252
* Sema7a*	2.8	0.0016	5.6	0.0006
* Nfatc1* (NFAT2)	1.6	0.0032	2.2	0.0001
* Itgb1* (integrin beta 1)	1.6	0.0000	1.6	0.0026
* Tpt1* (p21)	1.9	0.0029	2.5	0.0001

For each gene, the values represent the ratio between the expression level in the STHD‐01 group and that in the standard diet (SD) group (all gene expression levels are normalized to *Gapdh* expression). The *P‐*values are derived from *t*‐tests of the SD versus the STHD‐01 groups. See Table S1 for a description of the primers used for analyzing the expression of each gene.

### Evaluation of drug efficacy using the STHD‐01 models

**Figure 5:**
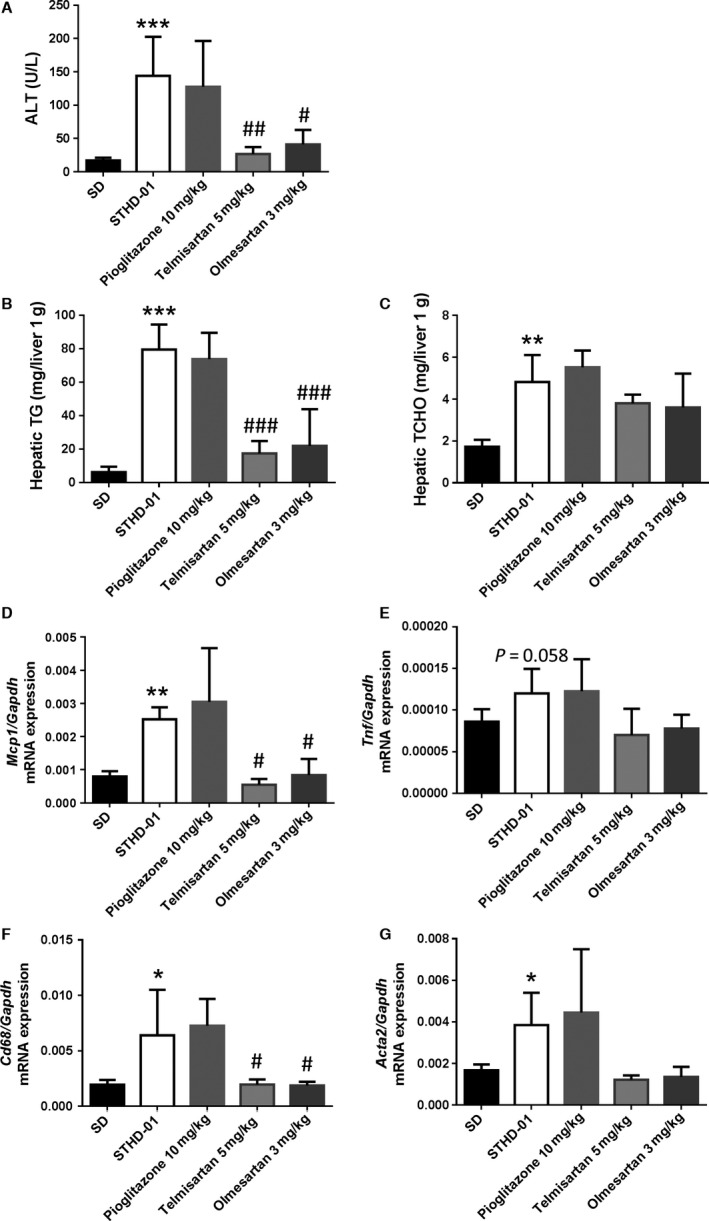
Alanine aminotransferase (ALT; hepatitis), hepatic triglycerides (TG; fat accumulation in the liver), hepatic cholesterol (TCHO) and gene expression in the livers of mice fed with steatohepatitis‐inducing high‐fat diet (STHD‐01) and administered pioglitazone, telmisartan, or olmesartan. ALT levels (A), hepatic TG levels (B), and hepatic TCHO levels (C), as well as expression levels of *Mcp1* (D), *Tnf* (E), *Cd68* (F), and *Acta2* (G) in the liver were determined after feeding with standard diet (SD) or STHD‐01 for 1 week (*n* = 4–5 in the SD and STHD‐01 groups). Bars represent the means ± standard errors. *P < 0.05, **P < 0.01, ***P < 0.001 compared with the SD group (Student's *t*‐test). ^#^ *P < *0.05, ^##^ *P < *0.01, ^###^ *P < *0.001 compared with the STHD‐01 group (Dunnett's test).

The angiotensin receptor blockers (ARBs) telmisartan and olmesartan, as well as ezetimibe and vitamin E significantly improved hepatitis/ALT elevation that was induced by short‐term STHD‐01 feeding (Table 3). Telmisartan in particular substantially and reproducibly inhibited plasma ALT elevation, inhibited accumulation of hepatic TG and TCHO, and increased hepatic expression of genes related to inflammation (*Mcp1*,* Tnf*,* Cd68*) and fibrosis (*Acta2*/*α*SMA) (Fig. 5). On the other hand, the effect of obeticholic acid was weak, while rolipram, pentoxifylline, pioglitazone, ursodeoxycholic acid, EPA, and BCAAs had no effect on hepatitis in this model.

**Table 3:** Inhibitory effects of drugs on alanine aminotransferase (ALT) elevation and hepatic triglyceride (TG) accumulation in mice fed with a steatohepatitis‐inducing high‐fat diet (STHD‐01) for 1 week

Drug evaluated	Dose	Inhibitory effect	
		ALT elevation	Hepatic TG accumulation
Telmisartan	5 mg/kg	++	+
Olmesartan	3 mg/kg	+	+
Ezetimibe	10 mg/kg	+	+
Vitamin E	500 mg/kg	+	−
Obeticholic acid	10 mg/kg	±	±
Rolipram	30 mg/kg	−	+
Pentoxifylline	50 mg/kg	−	−
Pioglitazone	10 mg/kg	−	−
Ursodeoxycholic acid	360 mg/kg	−	N.A.
Eicosapentaenoic acid	4000 mg/kg	−	−
Branched‐chain amino acids	4200 mg/kg	−	−

For each drug, the effect was evaluated in a different number of samples, as follows: telmisartan, *n* = 5–8 (multiple experiments); olmesasrtan, *n* = 5; ezetimibe, *n* = 5; vitamin E, *n* = 7; obeticholic acid, *n* = 8; rolipram, *n* = 6; pentoxifylline, *n* = 7; pioglitazone, *n* = 5; ursodeoxycholic acid, *n* = 5; eicosapentaenoic acid, *n* = 7; and branched‐chain amino acids, *n* = 8.

For each drug, the effect is expressed as STHD‐01 versus STHD‐01 +  drug.

Significance indicators: +, *P *< 0.05 or *P *< 0.01; ++, *P *< 0.001; ±, tendency; −, not significant; N.A., not analyzed (Student's *t*‐test or Dunnett's test).

**Figure 6:**
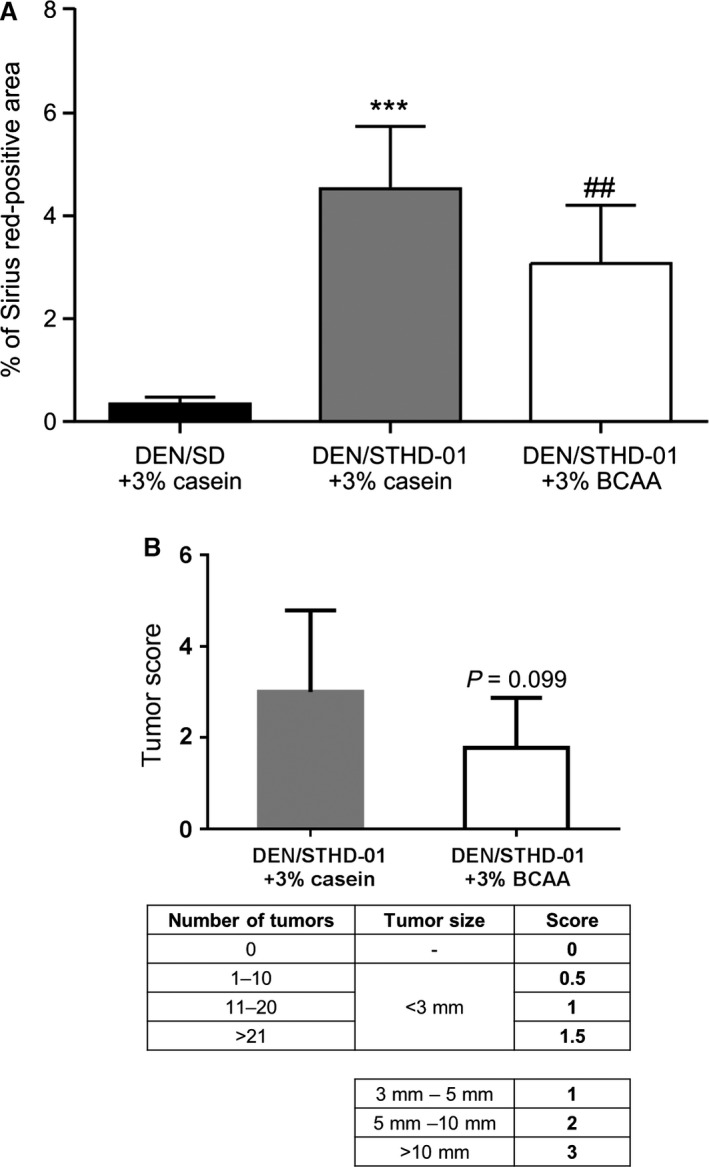
Effect of branched‐chain amino acids (BCAAs) on fibrosis in mice fed with steatohepatitis‐inducing high‐fat diet (STHD‐01) or standard diet (SD) for 30 weeks after diethylnitrosamine (DEN) pretreatment. The percentage of Sirius red‐positive areas indicating liver fibrosis (A, *n* = 9) and score for tumor size and number (B). Comparison between DEN/SD+3% casein and DEN/STHD‐01 + 3% casein, using the Student's *t*‐test (***P < 0.001). Comparison between DEN/STHD‐01 + 3% casein and DEN/STHD‐01 + 3% BCAA using the Student's *t*‐test (^##^ *P *< 0.01).

The effects of BCAAs, introduced with a formulation similar to that of a BCAA preparation used to treat hepatic cirrhosis in Japan (Livact^®^, EA Pharma, Tokyo, Japan) were examined in mice‐fed STHD‐01 for 30 weeks after DEN pretreatment. The BCAAs significantly decreased the percentage of the Sirius red‐positive area in the liver (Fig. 6A). The BCAAs tended to improve tumor scores, calculated according to the size and number of tumors confirmed macroscopically by necropsy, in mice‐fed STHD‐01 for 30 weeks after DEN pretreatment (Fig. 6B).

## Discussion

**Figure 7:**
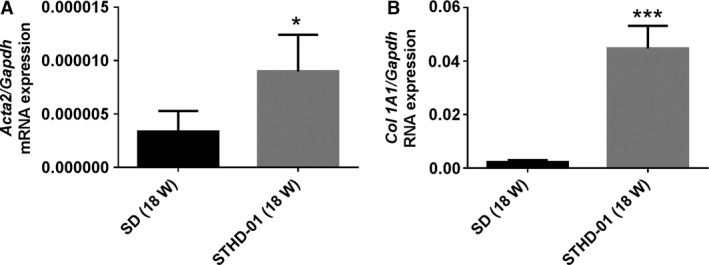
Expression levels for key genes in the livers of mice fed with steatohepatitis‐inducing high‐fat diet (STHD‐01) or standard diet (SD) for 18 weeks. Bars represent the means ± standard errors. *P < 0.05, **P < 0.01, ***P < 0.001 compared with the SD group (Student's *t*‐test).

Macrophages activated by cholesterol play a very important role in inducing steatohepatitis or arteriosclerosis (in the form of foam cells) (Khanna et al. 2013). Many studies have shown that cholesterol significantly increases the levels of serum leptin, interleukin‐6, fibrosis, and liver alpha‐smooth muscle actin (*α*‐SMA) in mice fed a solid diet containing 0.2% cholesterol with 30% fat, and that dysregulated hepatic cholesterol homeostasis as well as accumulation of liver free cholesterol are involved in the pathogenesis of NAFLD/NASH (Arguello et al. 1852; Mells et al. 2015). Hence, we propose the following mechanism of steatohepatitis induction by STHD‐01: First, TG and cholesterol accumulate in the hepatocytes. Overloading with fatty acids and cholesterol leads to injury to the hepatocytes. Subsequent activation of hepatic Kupffer cells and macrophages occurs as the saturated fatty acids and cholesterol leak from the hepatocytes, leading to TNF production and thereby direct liver damage. Furthermore, MCP‐1 induced by TNF causes accumulation of monocytes from the circulating blood in the liver, ultimately leading to chronic inflammation. In order to induce a state of steatohepatitis within a short time, it was necessary to mimic human dietary habits to an excessive extent. We therefore formulated STHD‐01 with a 5% cholesterol and 40% fat content. However, this was not a model of acute liver damage in which ALT is severely elevated by several thousand times, but rather a model of chronic, mild but persistent liver damage with an ALT elevation of only a few hundred times. The STHD‐01 short‐term model did in fact result in the accumulation of TG and cholesterol in the liver, causing liver damage (elevated ALT), and we confirmed elevated gene expression in the liver for *Cd68*, a marker for the activation of Kuppfer's cells and macrophages, as well as for *Tnf* and *Mcp‐1*. In mice‐fed STHD‐01 for 9 or 18 weeks, expression of fibrosis‐related genes (*Acta2*/*αSMA*,* Col1A1)* increased (Fig. 7). STHD‐01 augmented the expression of inflammation‐related genes such as *Tnf* and *Mcp1* in the liver, and enhanced TNF production from hepatic NPCs following LPS stimulation. It is interesting that a significant elevation of TNF production from NPCs was observed without LPS stimulation after 8 weeks of administering STHD‐01. We hypothesized that these NPCs are readily stimulated in vivo by lipotoxicity or endotoxin influx into the liver, and that intestinal permeability is increased. Liver fibrosis hence gradually develops during repeated cycles of inflammation‐induced damage and repair. In this STHD‐01 model, insulin and blood sugar levels did not change in the mice; however, blood concentrations of total bile acids increased (data not shown), and body weight gain tended to be slightly inhibited compared to that in the SD‐fed mice. Bile acids, which increase via the D2‐TGR5‐cAMP pathway, in turn upregulate cellular T3 levels, leading to increased energy consumption in the involved organs (Watanabe et al. 2006). This may explain, at least in part, why our model of long‐term SHTD‐01 feeding showed no insulin resistance and obesity; however, further investigation is required to confirm this hypothesis. Some patients, however, develop a so‐called “lean NAFLD/NASH” in the absence of metabolic syndrome associated with obesity and diabetes (Vos et al. 2011; Younossi et al. 2012). In this context, our model may be regarded as a lean NASH model, in light of further investigation of the clinical characteristics of lean NASH patients.

Previous reports suggested that ARBs improve hepatic inflammation and fibrosis in hypertensive patients with NAFLD or NASH (Georgescu et al. 2009; Musso et al. 2010; Paschos and Tziomalos 2012). Therefore, we decided that telmisartan would be a suitable positive control for this model; we also tested the ARB olmesartan. As expected, both ARBs tested were found to inhibit not only ALT elevation, but also hepatic TG and cholesterol accumulation. Moreover, we used pioglitazone to investigate the involvement of peroxisome proliferator‐activated receptor gamma (PPAR*γ*) activity in this short‐term NASH model. However, pioglitazone showed a negligible effect in this model, indicating that an alternative PPAR*γ*‐independent mechanism may be contributing to the efficacy of telmisartan. Some researchers have reported that telmisartan inhibits cytokine‐induced expression of VCAM‐1 in intravascular cells via suppression of NF‐*κ*B activation rather than PPAR*γ* activation (Nakano et al. 2009). We surmise that pioglitazone may inhibit the onset of hepatitis when fat accumulation into the liver is low and slow; however, pioglitazone‐induced pathways are unlikely to inhibit aggressive pathophysiological progression caused by excessive fat accumulation and hepatitis within as short a period of time as observed in the STHD‐01 feeding model.

In this model, ALT elevation was inhibited not only by telmisartan but also by the antioxidant vitamin E and ezetimibe, known to block the internalization of Niemann–Pick C1‐Like 1 protein and absorption of cholesterol from the small intestine. However, the anti‐inflammatory agents rolipram and pentoxifylline did not exhibit any apparent inhibitory effect on ALT elevation. We surmise that an anti‐inflammatory effect alone is not sufficient to suppress hepatopathy in our model. Furthermore, we did not observe any obvious efficacy of obeticholic acid/INT‐747. These results suggest that solely remedying lipid metabolism is not sufficient to produce efficacy in a model characterized by inflammation induced by excessive lipid loading, and drugs that reduce inflammation would need to be used along with those that correct lipid metabolism in order to effectively counter hepatopathy in our steatohepatitis model.

Interestingly, recent studies have shown that BCAA supplementation reduces the risk of hepatocellular carcinoma, prolongs survival of patients with cirrhosis, ameliorates fibrosis, and suppresses tumor growth in rat models (Cha et al. 2013; Kawaguchi et al. 2014). Furthermore, BCAAs are reported to suppress DEN‐induced liver tumorigenesis in obese and diabetic mice (Iwasa et al. 2010). We propose that BCAAs might induce suppression of fibrosis through inhibiting the expression of *Timp1* and *Tgfb1*, which are known to deactivate collagenolytic matrix metalloproteinases, rather than through improving lipid metabolism.

In conclusion, we characterized a novel experimental diet, STHD‐01, which induced NASH‐like phenotypes in mice. The most notable advantage of STHD‐01 is its ability to induce steatohepatitis in normal mice (C57BL/6J) within a period of time as short as 1 week. The use of this STHD‐01 feeding model for in vivo screening of drug candidates to improve inflammatory response after lipid accumulation in the liver should be considered. The long‐term STHD‐01 feeding model after DEN pretreatment may be useful for investigating post‐NASH conditions in humans.

## Conflict of Interest

All authors are employed by EA Pharma Co. Ltd., a wholly owned subsidiary of EA Pharma Co., Ltd.

## Supporting information




**Table S1.** Primer sequences used to analyze mRNA expression via RT‐PCR in liver samples from mice with diet‐induced steatohepatitis with fibrosis.
