# Supplementary material for: A novel diet‐induced murine model of steatohepatitis with fibrosis for screening and evaluation of drug candidates for nonalcoholic steatohepatitis
Source: Physiol Rep. 2016 Nov 8;4(21):e13016. doi: 10.14814/phy2.13016 (PMC5112494; doi:10.14814/phy2.13016)
Supplement: Supplementary file 1 — Table S1. Primer sequences used to analyze mRNA expression via RT‐PCR in liver samples from mice with diet‐induced steatohepatitis with fibrosis. [file PHY2-4-e13016-s001.docx]

SUPPLEMENTAL TABLE

**A novel diet-induced murine model of steatohepatitis with fibrosis for screening and evaluation of drug candidates for non-alcoholic steatohepatitis**

Chieko Ejima, Haruna Kuroda, and Sonoko Ishizaki

**Supplemental Table.**

Primer sequences used to analyze mRNA expression via RT-PCR in liver samples from mice with diet-induced steatohepatitis with fibrosis.

| Genes | Gene Symbol | Forward | Reverse |
| --- | --- | --- | --- |
| Inflammation |  |  |  |
| TNFalpha | *Tnf* | CACGCTCTTCTGTCTACTGAACTTC | ATGATCTGAGTGTGAGGGTCTGG |
| MCP-1 | *Mcp1* | CCACAACCACCTCAAGCACTTC | AATTAAGGCATCACAGTCCGAGTC |
| IL-6 | *Il6* | AACAACGATGATGCACTTGCAG | CTGAAGGACTCTGGCTTTGTCT |
| SPP1/osteopontin | *Spp1* | AATCTCACCATTCGGATGAGTCTG | TCTGTGGCATCAGGATACTGTTCA |
| IL-17 | *Il-17* | CCCTCAAAGCTCAGCGTGTC | AGGGTCTTCATTGCGGTGGAG |
| F4/80 | *Adgre1* | TTTGGCTATGGGCTTCCAGTC | TCAGCAACCTCGTGTCCTTGAG |
| NOS2 | *Nos2* | AGCTGAACTTGAGCGAGGAG | TGCCCCATAGGAAAAGACTG |
| Fibrosis |  |  |  |
| Procoll 1a1 | *Col1a1* | GAGCGGAGAGTACTGGATCG | AGACGGCTGAGTAGGGAACA |
| Alpha SMA | *Acta2* | AAACAGGAATACGACGAAG | CAGGAATGATTTGGAAAGGA |
| Timp1 | *Timp1* | CCCTTCGCATGGACATTTAT | GGCCATCATGGTATCTCTGG |
| TGFbeta | *Tgfb2* | GGAGAGCCCTGGATACCAAC | CAGGGTCCCAGACAGAAGTT |
| Hsp47 | *Serpinh1* | AAGGGAGACAAGATGCGAGA | TAGCACCCATGTGTCTCAGG |
| Premalignant lesions |  |  |  |
| EpCAM | *Epcam* | GCGGGGATTGTTGTCCTGGT | ACTCAGCACGGCTAGGCATTA |
| Cytokeratin8 | *Krt8* | ACTCACTAGCCCTGGCTTCA | TCTTCACAACCACAGCCTTG |
| Beta-Catenin | *Ctnnb1* | AGGGTGGGAATGGTTTTAGG | GTGGCAAAAACATCAACGTG |
| Conductin | *Axin2* | AGGAGCAGCTCAGCAAAAAG | CGTCCCAGATCTCCTCAAAA |
| Oxidative stress |  |  |  |
| Nrf2 | *Nrf2* | GAGTTGCCACCGCCAGGA | TGTCTTGCCTCCAAAGGATGTC |
| Heme oxygenase 1 | *Hmox1* | ATCAGCACTAGCTCATCCCAGACAC | AGAGAGAAGGCCACATTGGACAGAG |
| Endoplasmic reticulum stress |  |  |  |
| Activating transcription factor 4 | *Atf4* | AAGAGGTCCGTAAGGCAAGG | CAGCAAACACAGCAACACAA |
| Perk | *Eif2ak3* | CGTGACCCATCTGCACTAATTTGCAAG | AAGAATGACGCTATTCACTGCTGCAGAC |
| X-box binding protein 1 | *Xbp1* | TCAAATGTCCTTCCCCAGAG | GGTCCCCACTGACAGAGAAA |
| Chop | *Ddit3* | AGTCCATTTCAACCCGACTG | GGGCAGTTACGGAAGATGAA |
| Grp78 | *Hspa5* | CCGAGTGACAGCTGAAGACA | GCGCTCTTTGAGCTTTTTGT |
| TNF receptor-associated factor 2 | *Traf2* | TAGGTGGGTGCTCAGAAAGG | TAGCCGCTACCTCTTTTCCA |
| Metabolism |  |  |  |
| CYP7A1 | *Cyp7a1* | AGCAACTAAACAACCTGCCAGTACTA | GTCCGGATATTCAAGGATGCA |
| 3-phosphoglycerate dehydrogenase | *Phgdh* | ATGCTCAACGGAGCTGTCTT | AATCATAGTGGGCAGCATCC |
| PPARgamma | *Pparg* | AGACAACGGACAAATCACCA | CACCTCTTTGCTCTGCTCCT |
| Methylenetetrahydrofolate dehydrogenase | *Mthfd2* | GACCAGAGGAGCTGGAAGTG | CATTCCTCTTGGCCTGCTTA |
| UCP2 | *Ucp2* | CCTACAAGACCATTGCACGA | CATAGGTCACCAGCTCAGCA |
| Fibroblast growth factor 21 | *Fgf21* | AGAGAACTGCTGCTGGAGGA | CTGGTTTGGGGAGTCCTTCT |
| Other genes |  |  |  |
| p73alpha | *Trp73* | GCCCATCAAAGAGGAGTTCA | TCCCACTTCCAAGAGCAGTT |
| NFAT2 | *Nfatc1* | TGTGAGGAGTTGGCTCAGTG | TCGTTCGGTAAGTTGGGATT |
| Semaphorin 7A | *Sema7A* | ACTGGCCGAACAACTAATGG | AGCCCTAGTGAACCAGCAGA |
| Integrin beta 1 | *Itgb1* | TTTTGCAACACCAAGCTCAC | TTTCCAAACCTGCATGTGAA |
| p21 | *Cdkn1a* | TGCAATTGCTTCTGTTTGCT | GCAATGCTGAGATGCTCTTG |
